# Knowledge, Attitudes, and Practices (KAP) toward the COVID-19 Vaccine in Oman: A Pre-Campaign Cross-Sectional Study

**DOI:** 10.3390/vaccines9060602

**Published:** 2021-06-04

**Authors:** Sabria Al-Marshoudi, Haleema Al-Balushi, Adil Al-Wahaibi, Sulien Al-Khalili, Amal Al-Maani, Noura Al-Farsi, Adhari Al-Jahwari, Zeyana Al-Habsi, Maryam Al-Shaibi, Mahfoodh Al-Msharfi, Ahlam Al-Ismaili, Hood Al-Buloshi, Bader Al-Rawahi, Khalifa Al-Barwani, Seif Al-Abri

**Affiliations:** 1 Directorate-General for Disease Surveillance and Control (DGDSC), Ministry of Health, Muscat 393, Oman; haleema9292@yahoo.com (H.A.-B.); adilwahaibi@gmail.com (A.A.-W.); sulienkhalili18@gmail.com (S.A.-K.); amalsaifalmaani@gmail.com (A.A.-M.); nouralfarsi@gmail.com (N.A.-F.); athari-91@hotmail.com (A.A.-J.); nada000_000@hotmail.com (Z.A.-H.); mariamalshaibi@gmail.com (M.A.-S.); bderalrawahi4@gmail.com (B.A.-R.); salabri@gmail.com (S.A.-A.); 2 National Center for Statistic and Information (NCSI), Muscat 133, Oman; msharfi@ncsi.gov.om (M.A.-M.); aismaili@ncsi.gov.om (A.A.-I.); hbuloshi@ncsi.gov.om (H.A.-B.); ceo@ncsi.gov.om (K.A.-B.)

**Keywords:** COVID-19, Oman, vaccines, knowledge, attitude, practice, immunization programs, pandemics

## Abstract

Oman is globally acknowledged for its well-structured immunization program with high vaccination coverage. The massive spread of misinformation brought on by the COVID-19 pandemic, as well as the easy access to various media channels, may affect acceptance of a vaccine, despite the inherent trust in the local system. This cross-sectional study evaluated the knowledge, attitudes, and practice (KAP) in Oman toward COVID-19 vaccines. It included 3000 randomly selected adults answering a structured questionnaire via telephone. Participants were 66.7% Omani, 76% male, and 83.7% without comorbidities. Their mean age was 38.27 years (SD ± 10.45). Knowledge of COVID-19′s symptoms, mode of transmission, and attitudes toward the disease was adequate; 88.4% had heard of the vaccine, 59.3% would advise others to take it, 56.8% would take it themselves, and 47.5% would take a second dose. Males (CI = 2.37, OR = (2.00–2.81)) and Omani (CI = 1.956, OR = (4.595–2.397)) were more willing to be vaccinated. The history of chronic disease, source of vaccine knowledge, and education level were factors that affected the willingness to accept the vaccine. The Omani community’s willingness to take the COVID-19 vaccine can be enhanced by utilizing social media and community influencers to spread awareness about the vaccine’s safety and efficacy.

## 1. Introduction

The COVID-19 pandemic has imposed an enormous burden globally and locally by disrupting not only health services but social and economic systems. As per the World Health Organization (WHO) report for COVID-19 on 6 March 2021, there were 115,653,459 confirmed cases of COVID-19, including 2,571,823 deaths globally [1].

As a report from the National Center for Statistic and information (NCSI) 2021, Oman population is 4,471,148 as shown in annex1. Oman reported the first case of COVID-19 on 24 February 2020, and since the epidemic began, the national health system has evolved to impact the health and lives of the population directly or through different interventions implemented to control it [2,3]. Almost one year later, on 20 February 2021, a total of 139,692 confirmed cases of SARS-CoV-2 infection and 1555 cumulative deaths were reported in Oman by the Ministry of Health (MOH) [4]. The pandemic has resulted in 15% access to all causes of mortality [2].

Vaccination is considered a crucial advance in the field of public health, where it has succeeded in the eradication and control of many infectious diseases worldwide (e.g., smallpox, polio, and rubella) [5,6]. Oman has achieved remarkable improvement in the routine vaccination program over the past two decades and has been successful in interrupting the endemic transmission of vaccine-preventable diseases for more than a decade due to sustained, high-quality surveillance and immediate prevention and control measures [7,8]. Additionally, Oman eliminated poliovirus in 1994, and diphtheria has been eradicated in the sultanate since 1992. The routine vaccination program has an established system for the monitoring and follow-up of adverse events associated with vaccines. In 1996, Oman launch it is AFI surveillance program to address vaccine safety concerns [9,10]. In 2016, Oman achieved the highest effective vaccine management score (99%) for all criteria for all levels out of the 127 effective vaccine management assessments conducted globally in 90 countries [8]. In addition, Oman was certified as free from measles in 2019 [11].

The COVID-19 pandemic evolved worldwide, leading everyone to pursue solutions, including effective and safe vaccines to control the virus and minimize its impact [12]. By the end of 2020, globally vaccine manufactures have successfully concluded phase three trials of the three vaccines, giving the world hope for a quick end to the pandemic and promise for getting life in general back to normal. As few vaccines got the emergency approval for use, it became important in the process of deploying them to explore the community’s knowledge and attitude toward such intervention [13]. This will easily identify factors influencing vaccine hesitancy or acceptance, and hence it will help to deeply recognize the features that influence the public in adopting healthy practices and responsive behavior toward COVID-19 in general and, specifically, accepting vaccines [14].

Misinformation, spreading through multiple channels, could have considerable effects on the acceptance of a COVID-19 vaccine, even in populations where vaccine hesitancy is not an issue and the national immunization system is well-structured and trusted. This study was conducted to measure the level of willingness in the community to receive a potentially safe and effective new vaccine through a national KAP survey to assess vaccine knowledge, acceptance, and awareness in Oman’s society toward the COVID-19 vaccine.

## 2. Materials and Methods

### 2.1. Study Design and Participants 

This cross-sectional, phone-based survey was conducted at the national level with randomly selected individuals sourced from the national phone registry, representing all governorates of Oman. The interviews were conducted after verbal consent and in three languages: Arabic, English, or Urdu. If a participant did not understand any of the languages, they were excluded. The interviews were conducted in the period of 15–31 December 2020 with the help of the National Center for Statistics and Information (NCSI).

### 2.2. Sample Size Calculation

To determine the study sample size, we considered that >95% of the general population would present a high level of knowledge regarding COVID-19, evidenced by a recent study conducted in Oman evaluating the level of KAP toward COVID-19 in the 6 months preceding this study by the NCSI [15]. Therefore, based on the above study results, our study sample size was calculated as 2134 and set to be 3000 with a 3% margin of error and 95% CI. Forty-one thousand phone numbers from the entire country were selected randomly as a sample frame. Around 12,000 calls were made to reach the desired sample size of 3000 participants. Around 9000 calls were excluded from the study due to many reasons, such as no response, a busy signal, hanging up in the middle of the interview, unqualified respondents <18 years, and respondents who did not understand the language.

### 2.3. Questionnaire and Data Collection

A structured questionnaire (see Supplementary Materials) was designed to include 23 questions divided into 4 parts. The first part comprised questions regarding personal demographic information, such as nationality, age, gender, living governorate, education level, occupation, place of residence, and past medical history of chronic disease. The other 3 parts covered the knowledge and its sources, attitude, and practices related to the COVID-19 vaccine.

The questionnaire was developed in English and then translated into Arabic. After that, it was translated back into English to check for compatibility. The survey was reviewed many times in both languages by the research team and the NCSI. A piloted sample of 30 participants was conducted to test the reliability of the questions (test-retest) and the time needed to interview a participant. Trained data collectors were used to collect data from the NCSI daily. The data was reviewed by the supervisor from the NCSI daily to make sure that it was appropriately collected and saved.

Five questions regarding knowledge of COVID-19 were included. Answers consisted of scores from 0 to 5, of which 0 meant deficient and 5 meant very good information. The remaining classifications were good, satisfactory, and sufficient.

Scores for knowledge regarding the COVID-19 vaccine and news sources were coded as 0 = “No”, 1 = “Yes” and 2 = “Don’t know”. To calculate the score, a correct answer was computed as 1, and a wrong answer was computed as 0. Not answering or not knowing was considered neutral and calculated as 0.

The attitude section included three items evaluating the level of concern regarding the vaccine. The response options were 0 = “No”, 1 = “Yes”, and 2 = “Don’t know”, and if the participant had any concerns, the reason for concern was asked. Additionally, responders were asked if they would advise family and friends to take the vaccine.

The practice section included four items used to assess the level of agreement with the following statement: if the responder was willing to take both doses of the vaccine and would inform the nearest institution regarding any side effects. If the participant refused or was not sure if they would report side effects, the reason for this was asked. The response options were 0 = “No”, 1 = “Yes”, and 2 = “Don’t know”.

### 2.4. Statistical Analysis

Data were analyzed using the ‘survey’ package in R software (R-package version 4.0., R Foundation, https://www.r-project.org/ (accessed on 25 February 2021)). Categorical variables were presented as weighted proportions. This weighting was done using ‘sex and age group’. Scales such as the ‘rating of knowledge’ were described as continuous variables using a weighted mean and standard deviation (SD). Univariate logistic regression was used to investigate factors affecting the willingness to vaccinate. A *p*-value of <0.05 was considered to be statistically significant.

## 3. Results

### 3.1. Sociodemographic Characteristics

A total of 3000 participants completed the phone-based questionnaire, and among them, 66.7% were Omani. The mean age of the respondents was 38.27 years (SD + −10.45). Males accounted for 76% of the respondents. The highest number of participants was from the Muscat Governorate (28.22%), and the lowest was from the Al Wusta Governorate (1.1%). These results are proportional to the governorate populations. Most of the participants were pre-secondary school certificate holders (54.6%). Almost three-quarters (71.4%) of the participants were working, and 40.6% of them worked in the private sector. Out of the total participants, 83.7% had no previous comorbidities. The baseline characteristics of the responders are shown in Table 1.

### 3.2. Knowledge of COVID-19 Disease

As for the knowledge, there were no differences in the means of the participants who knew about COVID-19′s symptoms, modes of transmission, and the highest-risk group that could get the disease and its complications (7.8, 7.9, and 7.8), respectively. Likewise, the means of those who knew how to act when they had symptoms of COVID-19 and how to protect themselves from the disease were 8.3 and 8.4, respectively. Table 2 shows the overall knowledge level related to COVID-19.

### 3.3. Knowledge of the COVID-19 Vaccine and the Scores of the Sources of Information

The majority of the participants (88%) had heard of COVID-19, and the most common source of information was social media (67%), followed by television (56%). Most participants (52%) thought that vaccines could protect them from contracting COVID-19, and 42% believed that patients could not contract COVID-19 after taking the vaccine. Regarding the technical issues related to the COVID-19 vaccine, 45% of participants knew that the vaccines would be given in two doses. Additionally, 29% of them thought that the vaccine could not be given to a person who had symptoms of the disease at the time of vaccination, and 44% believed that the vaccine could be given to a person with a previous history of contracting COVID-19. Almost one-quarter (26%) of them knew about the side effects of the vaccine, and 17% of the participants thought the vaccine would be safe but with some side effects. The overall knowledge related to the COVID-19 vaccine and sources of information is shown in Table 3.

### 3.4. Attitudes toward the COVID-19 Vaccine

**Figure 1:**
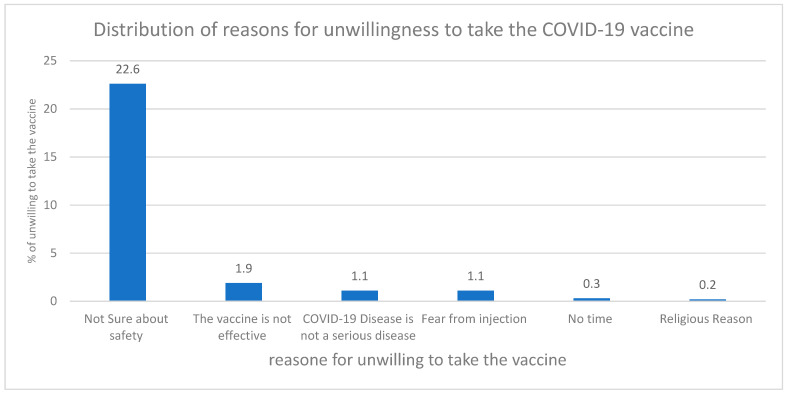
Distribution of reasons for unwillingness to take the COVID-19 vaccine.

The majority of the responders (59.3%) did not have any concerns regarding the vaccine, and they would advise their family and friends to get it. Among the participants, around 34% of those had concerns regarding the vaccine; the main drive of their concern was related to personal doubts about the efficacy and safety of the vaccine, as shown in Figure 1.

### 3.5. Practices toward the COVID-19 Vaccine

**Figure A1:**
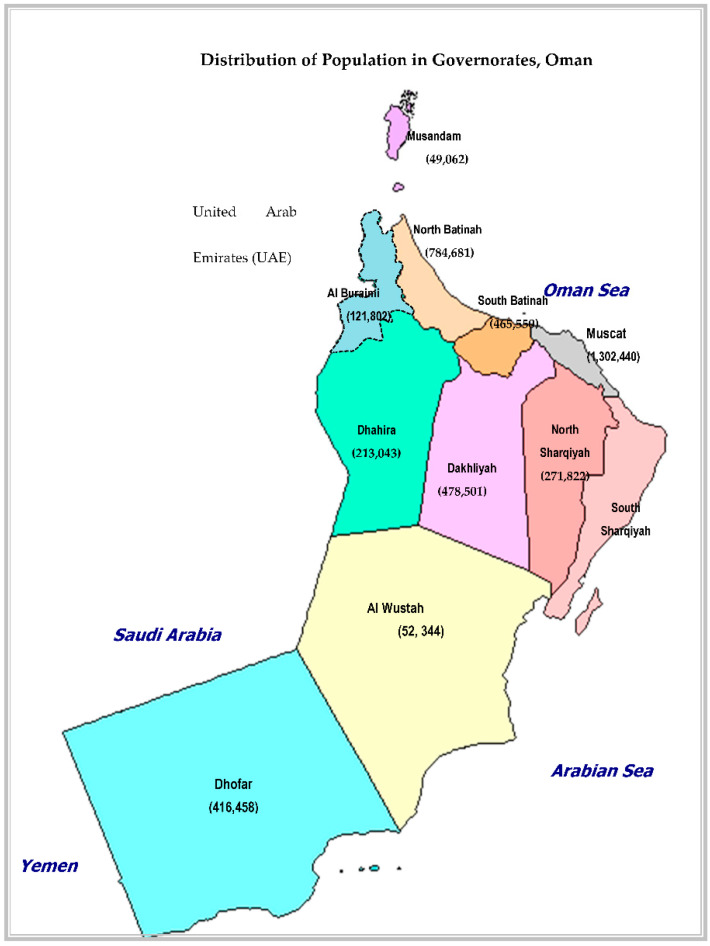
Distribution of Omani population according to governorates, Year 2021, Oman.

Over half of the responders (57%) were willing to take the vaccine, and 84% of the ones willing to take the vaccine would commit to taking the second dose as well. In addition, 97.5% of them would report the side effects to the health institute if any side effects were experienced. However, the reason 60% of those were not willing to get the vaccine was due to uncertainty about the vaccine’s safety. Figure A1 demonstrates the distribution of reasons for the unwillingness of taking the COVID-19 vaccine.

### 3.6. Factors Associated with Willingness to Take the COVID-19 Vaccine among the Public in Oman

Males were more willing to take the vaccine compared to females (CI = 2.37, OR = 2.00–2.81), and non-Omani people were more willing to be vaccinated than Omanis (CI = 0.49, OR = 0.42–0.57). In addition, participants with a history of chronic disease were more willing to take the vaccine in comparison with healthy people (CI = 1.3, OR = 1.06–1.58), especially participants with diabetes mellitus (DM) (CI = 1.69, OR = 1.28–2.23). On the other hand, pregnant women were less willing to get the vaccine (CI = 0.13, OR = 0.03–0.37). Those who had heard about the vaccine from their friends claimed to be more likely to take the vaccine compared with those who heard about it from different sources (CI = 1.38, OR = 0.99–1.94). In addition, those who believed the vaccine to be safe with some side effects were more inclined to take the vaccine than others (CI = 4.9, OR = 4.01–6.03). Furthermore, illiterate people were more willing to be vaccinated compared with people with post-secondary school or higher education levels (CI = 0.75, OR = 0.57–0.99). In addition, working participants were more willing to be vaccinated compared with those who were not working (CI = 1.72, OR = 1.47–2.02).

The multivariable analysis performed to investigate the significant factors of univariate analysis, after adjustment for sex, region, nationality, level of education, working state, and history of chronic disease, as shown in Table 4. It was show that Omani people were more willing to take the vaccine than non-Omani people were; otherwise, other results were similar to the univariate. 

## 4. Discussion

Our findings show that 57% of our sample population were willing to take the vaccine against COVID-19 as soon as they were targeted, based on the national immunization plan, and 59.3% would advise their family and friends to get this vaccine. This vaccination willingness in our community adds to the unsurprisingly wide scale of variation between the countries. A study done by Detoc et al. [16] showed that 77% of participants would agree to take the vaccine. In terms of comparing attitudes globally, studies have shown divergence among countries. Abdul et al. showed the scale of willingness to take the COVID-19 vaccine as soon as it became available in North America, South America, and Europe. The highest proportion of positive responses came from Panama (87.44%), and the lowest proportion of responses came from Russia (51.34%). In addition, the scale of strong compliance to COVID-19 vaccination as soon as it reached Africa, Asia, and Australia showed that Australia had the highest proportion of positive responses (92.88%), and the lowest proportion of responses was seen in Egypt (43.55%). This data clearly shows how great the discrepancies are between countries when it comes to the willingness to take the vaccine [14].

Our study showed that males were more willing to be vaccinated compared with females, a finding illustrated and supported by other evidence reported in the literature [17,18,19]. This is believed to be because women are more concerned about adverse side effects of the vaccine than contracting COVID-19 [20]. In a different light, Al Hanawi et al. stated that in the KAP survey about COVID-19 conducted among the Saudi community, females were found to be more knowledgeable and have positive practices and attitudes toward nonpharmaceutical preventive interventions [21].

What is more, in our study, pregnant women were less willing to receive the vaccine, which is supported with a study women in the US and Russia, who consistently expressed lower acceptance and confidence in the COVID-19 vaccine’s safety and effectiveness. This is in contrast with study conducted by Malia et al. 2021 in sixteen countries, with the results for acceptance in India, the Philippines, and Latin America being above 60% among pregnant women. This is in contrast with women in the US and Russia, who consistently expressed lower acceptance and confidence in the COVID-19 vaccine’s safety and effectiveness. The top reasons for mothers to be unwilling to be vaccinated for COVID-19 were that they were concerned that approval of the vaccine would be rushed for political reasons (39.8%), they would like to see more safety and effectiveness data among them (32.7%), and they believe that the vaccine is not safe and could have harmful side effects (28.4%) [22].

Our study found that participants with a history of chronic disease reported being more committed to getting the vaccine than healthy people. Those with diabetes mellitus were more likely to get the vaccine than those with other chronic diseases, probably due to the well-recognized high risk of developing severe complications if they contract COVID-19 [16].

This survey showed that people with low literacy were more willing to get the vaccine compared with people with post-secondary school or higher education levels. This contradicts what was shown in other studies, which found that people with low educational levels were more likely to refuse vaccination [23,24,25]. The participants in our study who believed the vaccine to be safe with some side effects were only 17%, a considerably low percentage due to the misinformation and rumors related to the vaccine. Because the pandemic started in December 2019 and so much was unknown at its inception, misinformation evolved alongside facts about the disease, and this affected perceptions worldwide [26]. A similar effect was recognized in another study on Congolese healthcare workers, which showed that misinformation regarding COVID-19 directly affected even the healthcare workers [6]. This indicates that even educated people can be affected directly by rumors, as was also stated by Reuben et al. [27].

Having no concerns about vaccine safety and increased awareness regarding vaccine side effects would make people more likely to take the vaccine, and this would be intensely associated with vaccine acceptance [20]. Acceptance was supported by a study conducted in 19 countries by Lazarus et al., where 71.5% of responders reported that they would take a vaccine if it were proven to be safe and effective [26].

One purpose of our KAP survey was to identify the knowledge–behavior gap and consequently determine the effective intervention and implement it [28]. Knowledge regarding COVID-19 influences the intake of vaccines, as shown in our study. Individuals who showed high knowledge regarding the disease, its symptoms, and modes of transmission indicated that they were more intent to be vaccinated against COVID-19. This is similar to findings from other studies which showed that high knowledge was significantly associated with a more positive attitude and perception [28,29].

Our study’s findings illustrates that 88% of participants heard about the COVID-19 vaccine, and this was in line with similar studies conducted in 19 countries demonstrating that knowledge significantly affects precautionary measures through the effectiveness of belief and had a direct effect on attitudes [14]. 

The most common sources of information for vaccines in our study were social media (67%) and television (56%). Certainly, trust is an essential and modifiable factor in the successful uptake of a COVID-19 vaccine. Our findings were supported by Ali et al. and Al hanawi MK et al. in a recent KAP study conducted in Saudi Arabia and Egypt, which showed high perception results regarding the main source of information related to the COVID-19 pandemic: social media (85.8% and 80.0%, respectively) and television (35.7% and 80.8%, respectively) [21]. In addition, our findings showed that friends were the most trusted source of information, highlighting the peer effects on the willingness to get vaccinated. The effects of media and peer influence in the acceptance of vaccination are supported by the fact that Omanis are more willing to be vaccinated against COVID-19 than non-Omanis, and this is probably due to the larger number of Omani participants in the survey compared with non-Omanis. This corresponds to findings from a study by Nguyen showing that the understanding of how the vaccine works will reduce the pandemic’s consequences [24], especially if that understanding is shared between people. The thing that will promote vaccine confidence to the community is tackling the rumors and adapting information from its official sources.

This study is considered to be the largest population-based study conducted in Oman to investigate the willingness of participants to get a COVID-19 vaccine, involving a randomized stratified population regardless of nationality. Additionally, this study will help decision-makers to modify strategic vaccination plans and identify the target groups for vaccination. In addition, assessing the information regarding the vaccine will help to target information inadequacy among the community and specific groups, tackle rumors, and design appropriate strategic management plans to enhance the quality of awareness to accept the vaccines.

Our study was conducted in a short period of 2 weeks, and this could be a limitation. In addition, selecting people by phone number could cause a bias, as selecting people with phones may exclude many others who do not have phones. Our study targeted the community at large while not representing the healthcare workers on a large scale; thus, there was a limited assessment of one of the greatest influencers to overall public opinion and information. In addition, language was a barrier, because the interviewer used only three languages—Arabic, English, and Urdu—where others spoke another language. Furthermore, assessing the acceptance level of the vaccine based on phone call surveys was influenced by many other factors apart from personal willingness, such as the timing of the call and the mood of the responder at the time of the call. Consequently, reporting probable intent to vaccinate does not represent a precise indicator for acceptance [14].

Research efforts have generated potentially effective strategies to improve vaccine acceptance and uptake, which go beyond traditional information campaigns aspiring to change behaviors by strengthening knowledge. Information on its own has shown a limited impact on facilitating vaccination uptake, while evidence on promoting vaccination in general is useful in the context of the current pandemic. The acceptance and uptake of COVID-19 vaccines present an unprecedented challenge [30]. Such approaches should be implemented by the MOH to achieve higher vaccination coverage. Some methods can be adopted to increase the uptake of the vaccine, such as free vaccination for all the targeted groups, equality and equity in the distribution of the vaccine among target groups by high-risk and priority groups, and by launching a national media campaign to promote the vaccine. Other methods to encourage community acceptance and encourage people to take the vaccine are, for example, to use public endorsements from government and community leaders and to create a waiting list for people who are not in the announced target groups but are interested in getting the vaccine. Other tactics include promoting the role of behavioral science in attempts to reach each group appropriately and promoting community partnership by holding regular teleseminars to receive and respond directly to questions from the general public. Moreover, increasing vaccine acceptance means investing more in conducting professional training in community engagement and risk communication skills by specialists in this field.

## 5. Conclusions

With a 34% rate of COVID-19 vaccine refusal by the participants, influenced mainly by friends and social media, the government should adopt innovative risk communication methods to reach all the resistant strata of the population highlighted in this study.

## Figures and Tables

**Table 1:** Sociodemographic information of the total number of participants (3000).

Section A: Demographic Characteristic	Total No.	Weighted Mean (SD) and Percentage	Total No. and Proportion of General Population
Age mean (SD)	3000	27.38 (45.10)	4,471,148
Gender			
Male	2280	76.01%	61.3%
Female	720	23.99%	38.7%
Governorate			
Muscat	846	28.22%	29.1%
Dhofar	282	9.41%	9.3%
Musandam	36	1.20%	1.1%
Al Buraymi	84	2.79%	2.7%
Ad Dakhiliyah	322	10.74%	10.7%
N. Batinah	537	17.90%	17.5%
S. Batinah	320	10.66%	10.4%
S. Sharqiyah	210	7.00%	7.1%
N. Sharqiyah	179	5.98%	6.1%
Al Dahirah	149	4.96%	4.8%
Al Wusta	34	1.14%	1.2%
Nationality			
Omani	2000	66.67%	61.1%
Non-Omani	1000	33.30%	38.9%
Education			
Illiterate	239	8.04%	
Pre-secondary school	1639	54.64%	
Post-secondary school and higher education	1118	37.32%	
Working			
Yes	2142	71.39%	67.4%
Government	819	27.30%	
Private	1217	40.57%	
Family	102	3.40%	
Other	3	0.12%	
Not working	857	28.60%	32.5%
Job seekers	212	7.10%	
Housewife	295	9.80%	
Full-time student	101	3.40%	
Retired	204	6.80%	
Unable to work	12	0.40%	
Unwilling to work	7	0.20%	
My work has stopped due to the pandemic	26	0.90%	
History of chronic disease			
No	2512	83.70%	
Yes	488	16.30%	

**Table 2:** Knowledge levels of the respondents regarding COVID-19 (N = 3000).

Section B: Knowledge of COVID-19 out of 3000 Responders	Weighted Mean	Weighted SD
Knowledge rating regarding awareness of COVID-19 symptoms	7.80	1.96
Information rating about the transmission methods of COVID-19	7.90	2.05
Knowledge rating regarding the correct way to act in the event you have symptoms of COVID-19	8.27	1.98
Rating knowledge about the most high-risk group and complications of COVID-19	7.76	2.18
Rating knowledge for reducing the risk of developing COVID-19	8.40	1.86

**Table 3:** Knowledge levels of the respondents regarding the COVID-19 vaccine and the sources of information (N = 3000).

Section C: Knowledge Regarding the COVID-19 Vaccine and News Source Scores out of 2652 Responders	N	Weighted%
Heard about the COVID-19 vaccine	2645	88
Think that the COVID-19 vaccine is safe with some side effects	453	17
Think that the COVID-19 vaccine protects from getting COVID-19	1363	52
Think it not possible to get COVID-19 even after taking the COVID-19 vaccine	1097	42
Think it possible to give the COVID-19 vaccine to a person with a history of COVID-19	1164	44
Think it is not possible to give the COVID-19 vaccine to a person suffering from COVID-19	763	29
Think that fever, slight swelling, and redness at the injection site are the side effects of the COVID-19 vaccine	684	26
Think that the COVID-19 vaccine is given in 2 doses	1192	45

**Table 4:** Factors associated with an increased willingness to take the COVID-19 vaccine among the public in Oman (N = 3000).

Factors	Sub-Category	Crude Odds Ratio (95% CI)	Adjusted Odds Ratio (95% CI)
	39–40	Ref.	
Age group	18–30	0.96 (0.81–1.13)	
	51–65	1.17 (0.92–1.49)	
	Over 65	1.87 (0.80–4.87)	
Sex	Male	Ref.	
	Female	2.37 (2.00–2.81)	0.493 (0.403–0.602)
Region	MUSCAT	Ref.	
	Al Dahirah	1.41 (1.03–1.94)	1.577 (1.132–2.197)
	Al Dakhiliyah	1.27 (0.99–1.64)	1.485 (1.135–1.942)
	Al Wusta	1.10 (0.62–1.95)	0.885 (0.493–1.588)
	Dhofar	0.78 (0.59–1.04)	0.756 (0.566–1.01)
	Musandam	0.94 (0.58–1.54)	0.967 (0.585–1.598)
	Al Buraymi	1.26 (0.82–1.94)	1.294 (0.83–2.018)
	N. Batinah	1.03 (0.81–1.33)	1.155 (0.888–1.502)
	N. Sharqiyah	0.87 (0.65–1.17)	1.009 (0.744–1.37)
	S. Batinah	1.05 (0.8–1.37)	1.201 (0.907–1.591)
	S. Sharqiyah	0.76 (0.57–1.02)	0.833 (0.613–1.131)
Nationality	Omani	Ref	
	Non-Omani	0.49 (0.42–0.57)	1.956 (1.5952.397)
Level of education	Illiterate	Ref	
	Post-secondary school and higher education	0.75 (0.57–0.99)	0.992 (0.73–1.349
	Pre-secondary school	0.89 (0.67–1.16)	1.071 (0.802–1.431)
Working	Not working	Ref	
	Working	1.72 (1.47–2.02)	
Sector where working	Government	1.16 (0.92–1.46)	0.921 (0.716–1.186)
	Private	1.7 (1.36–2.12)	0.871 (0.663–1.144)
	Business	1.11 (0.73–1.69)	0.618 (0.391–0.976)
	Other	0.68 (0.52–0.89)	0.666 (0.504–0.879)
History of chronic disease	Healthy	Ref	
	Chronic Disease	1.30 (1.06–1.58)	1.415 (1.147–1.746)
From those who have a chronic disease *	DM	1.69 (1.28–2.23)	
	HTN	1.27 (0.95–1.71)	
	Obesity	1.15 (0.19–8.73)	
	Immunocompromised	0.57 (0.11–2.61)	
	Renal disease	0.92 (0.28–3.19)	
	Heart disease	0.82 (0.38–1.78)	
	Asthma	0.93 (0.5–1.73)	
	Pregnant	0.13 (0.03–0.37)	
Knowledge regarding COVID-19	Rating COVID-19 symptom awareness	1.06 (1.02–1.09)	
	Rating COVID-19 transmission methods awareness	1.04 (1.00–1.07)	
	Rating COVID-19 attitude if showing symptoms awareness	1.00 (0.96–1.03)	
	Rating COVID-19 high-risk group complications awareness	1.00 (0.97–1.03)	
	Rating ways to reduce the risk of developing COVID-19 awareness	1.01 (0.97–1.05)	
Knowledge regarding the COVID-19 vaccine			
Have you heard about the vaccine?	No	Ref	
	Yes	1.10 (0.87–1.39)	
	Don’t know	1.31 (0.68–2.59)	
Source of information	MOH	1.20 (0.94–1.54)	
	International health organization	1.14 (0.81–1.61)	
	Friends	2.07 (1.54–2.8)	
	Neighbors	1.54 (0.90–2.75)	
	Social gathering	1.38 (0.99–1.94)	
	Health care worker	1.19 (0.66–2.21)	
	Social media	0.85 (0.73–0.98)	
	Newspaper	1.07 (0.75–1.53)	
	Radio	0.96 (0.76–1.22)	
	Television	1.02 (0.88–1.17)	
Is the COVID-19 vaccine safe?	It is safe and without side effects	1.13 (0.92–1.39)	
	It is safe and with some side effects	4.90 (4.01–6.03)	
	It is not safe and with obvious side effects	0.15 (0.11–0.20)	
	Don’t know	0.66 (0.57–0.76)	
Can the COVID-19 vaccine protect you from getting COVID-19?	No	Ref	
	Yes	8.20 (6.51–10.37)	
	Don’t know	2.57 (2.05–3.24)	
Can contract COVID-19 even after taking the COVID-19 vaccine?	No	Ref	
	Yes	0.44 (0.36–0.54)	
	Don’t know	0.42 (0.34–0.51)	
Can the COVID-19 vaccine be given if have a history of COVID-19 infection?	No	Ref	
	Yes	2.45 (2.02–2.97)	
	Don’t know	1.50 (1.24–1.82)	
Can the COVID-19 vaccine be given while suffering from COVID-19?	No	Ref	
	Yes	1.81 (1.50–2.18)	
	Don’t know	1.19 (0.99–1.43)	
What are the side effects that the COVID-19 vaccine can cause?	Fever, slight swelling, and redness at the injection site	Ref	
	No side effects	4.41 (2.89–6.99)	
	Don’t know	0.72 (0.60–0.86)	
How many doses of the COVID-19 vaccine should you receive?	One dose	Ref	
	Two doses	1.45 (1.13–1.85)	
	Don’t know	0.87 (0.68–1.10)	
Attitude			
Do you have any concerns about receiving the COVID-19 vaccine?	No	Ref	
	Yes	0.08 (0.07–0.10)	
	Don’t know	0.18 (0.13–0.25)	
Concern due to	Yes	Ref	
	Media effect	0.31 (0.21–0.46)	
	Personal effect	0.12 (0.10–0.15)	
	Personal doubts about effect and safety	0.29 (0.17–0.46)	

* The reference level is absent.

## Data Availability

Data in this study are available on request.

## Supplementary Materials

The following are available online at https://www.mdpi.com/article/10.3390/vaccines9060602/s1. Figure S1: Scoring of the source of information for COVID-19 Vaccine, Figure S2: Distribution of the participants who have a concern about COVID-19 vaccine based on drive of concern.
