# Supplementary material for: Knowledge, Attitudes, and Practices (KAP) toward the COVID-19 Vaccine in Oman: A Pre-Campaign Cross-Sectional Study
Source: Vaccines (Basel). 2021 Jun 4;9(6):602. doi: 10.3390/vaccines9060602 (PMC8228881; doi:10.3390/vaccines9060602)

Appendix A

Supplementary Figure S1. Scoring of the source of information for COVID-19 Vaccine

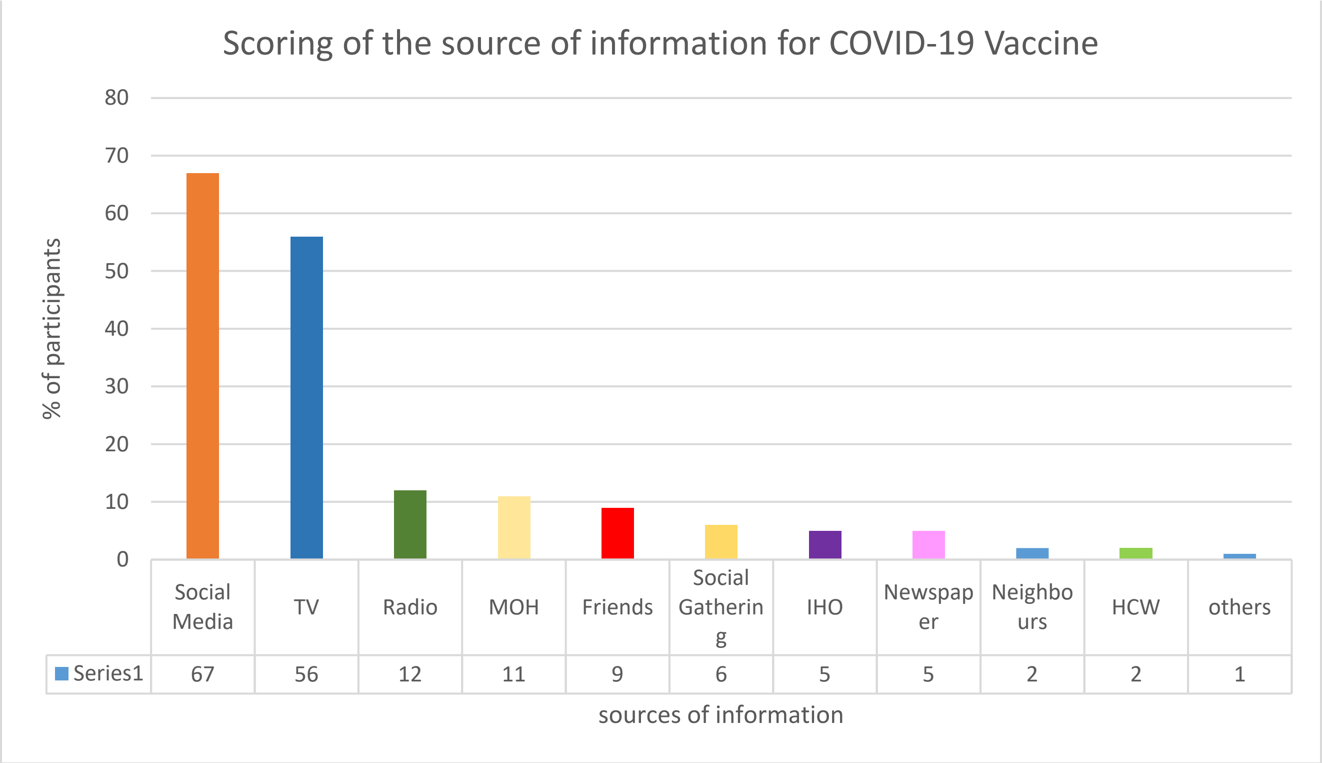

Supplementary Figure S2. Distribution of the participants who have a concern about COVID-19 vaccine based on drive of concern

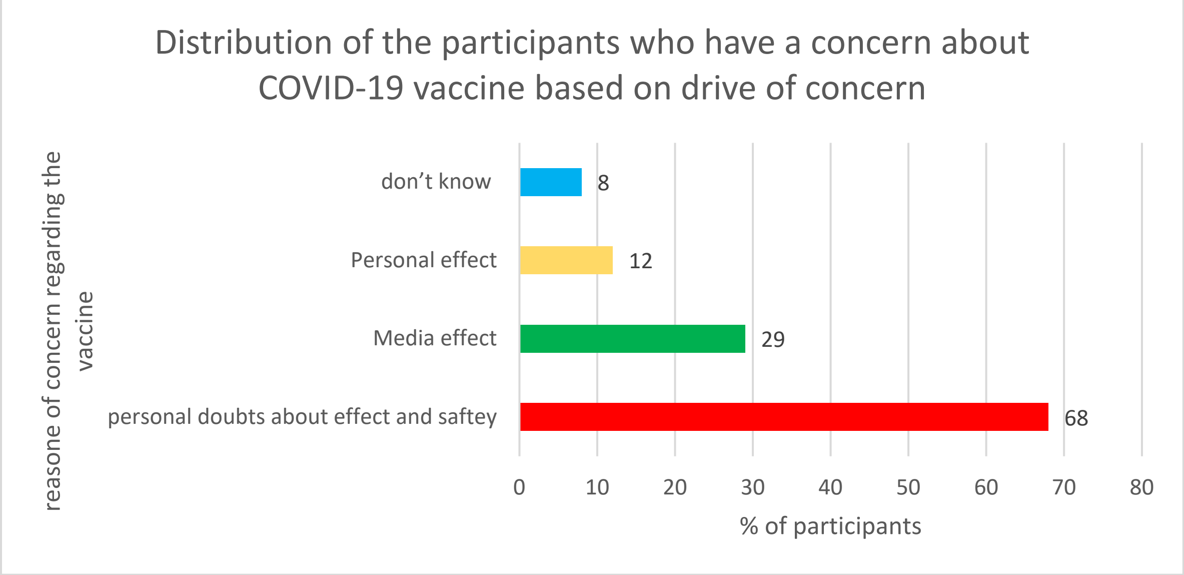

Supplement: Supplementary file 1 [file vaccines-09-00602-s001.zip › vaccines-1194496-supplementary.pdf]
